# The Physician’s Pocket Account Book

**Published:** 1911-04

**Authors:** 


THE PHYSICIAN'S POCKET ACCOUNT BOOK. By J. J. Taylor, M. D. 212 pages. Leather. Price, $1.00 postpaid. J. J. Taylor, Publisher, 4105 Walnut Street, Philadelphia, Pa.

The especial feature of this book is a system of accounts whereby each transaction can be recorded in a moment's time in plain language, so that it is strictly legal as evidence in court without personal explanation, and so arranged that any patron's account can be ascertained on demand without any posting. There is only one entry of each transaction, and this in such a form that no posting is ever required. It saves time, labor and worry, and insures that your accounts are always up to date, so that you can send statements out every month without any delay and can inform any patron, wherever you may meet him, of the exact state of his account. This feature alone in the course of a year will secure payments for you—that would otherwise be missed—sufficient to buy your account books for a whole lifetime. It is the simplest, quickest and easiest legal account system on the market.

The book also has some easy and practical directions for billing and collecting, some excellent business and legal hints, some valuable forms for emergency use, such as "dying declarations," "form for wills," etc., an average medical and surgical fee bill, besides miscellaneous tables, clinical directions, etc. Having a good cash account department and various clinical records—vaccinations, deaths and confinements—it forms a complete year-book for the physician's pocket.

For those who prefer to keep their accounts at the desk, the same system has been enlarged into a desk-size book of 400 large-sized pages, the price of which is only $5.00 per copy.

Readers of the "Red Back" will find this book a great convenience and a labor-saving and money-saving device. Get one, by all means.—D.

